# Hydrophilic Polyhedral Oligomeric Silsesquioxane, POSS(OH)_32_, as a Complexing Nanocarrier for Doxorubicin and Daunorubicin

**DOI:** 10.3390/ma13235512

**Published:** 2020-12-03

**Authors:** Kinga Piorecka, Anna Janaszewska, Marta Majkowska, Monika Marcinkowska, Jan Kurjata, Slawomir Kazmierski, Ewa Radzikowska-Cieciura, Bartlomiej Kost, Przemyslaw Sowinski, Barbara Klajnert-Maculewicz, Wlodzimierz A. Stanczyk

**Affiliations:** 1 Centre of Molecular and Macromolecular Studies, Polish Academy of Sciences, Sienkiewicza 112, 90-363 Lodz, Poland; jkurjata@cbmm.lodz.pl (J.K.); kaslawek@cbmm.lodz.pl (S.K.); eradziko@cbmm.lodz.pl (E.R.-C.); kost@cbmm.lodz.pl (B.K.); przem_so@cbmm.lodz.pl (P.S.); was@cbmm.lodz.pl (W.A.S.); 2 Department of General Biophysics, Faculty of Biology and Environmental Protection, University of Lodz, Pomorska 141/143, 90-236 Lodz, Poland; marta.k.majkowska@gmail.com (M.M.); monika.marcinkowska@biol.uni.lodz.pl (M.M.); barbara.klajnert@biol.uni.lodz.pl (B.K.-M.)

**Keywords:** anti-cancer complexes, non-covalent systems, polyhedral oligomeric silsesquioxanes, anthracyclines, in vitro viability studies

## Abstract

A novel strategy, recently developed by us, to use polyhedral oligomeric silsesquioxanes (POSS) as an anti-cancer drug carrier is presented. Anthracycline:POSS complexes were prepared by simple co-addition of doxorubicin (DOX) or daunorubicin (DAU) with hydrophilic POSS(OH)_32_. Co-delivery of POSS and anthracyclines led to higher anti-cancer activity towards HeLa (cervical cancer endothelial) and MCF-7 (human breast adenocarcinoma) cell lines. The obtained supramolecular hybrid complexes were characterised by nuclear magnetic resonance (NMR) spectroscopy (nuclear Overhauser effect spectroscopy [NOESY] and homonuclear correlation spectroscopy [COSY]), Fourier transform infrared spectroscopy (FTIR), and dynamic light scattering (DLS). The two-dimensional (2D) NOESY spectra of the complexes showed the cross-correlation peaks for hydroxyl groups of POSS (~4.3–4.8 ppm) with OH groups of DOX and DAU. FTIR showed that hydroxyl group of POSS can interact with amine and hydroxyl groups of DOX and DAU. The viability of HeLa and MCF-7 was analysed with the MTT assay to evaluate the cytotoxicity of free DOX and DAU and the relevant complexes with POSS at different molar ratios. At a low DOX concentration (2.5 µM), for molar ratios 1:1, 1:4, and 1:8 (POSS:DOX), the complexes showed two and three times higher cytotoxicity towards HeLa and MCF-7 cells, respectively, than DOX itself after both 24- and 48-h incubation. The 1 µM concentration for a 1:4 POSS:DOX molecular ratio and the 2.5 µM concentration for all complexes were more toxic towards MCF-7 cells than free DOX after 48-h incubation. In the case of POSS:DAU complexes, there was higher toxicity than that of free drug after 48-h incubation. It can be concluded that the formation of non-covalent complexes increases toxicity of anthracycline drugs towards Hela and MCF-7 cells. The novel complexes are inexpensive to prepare and more effective than free drugs at low systemic toxicity.

## 1. Introduction

**Figure 1:**
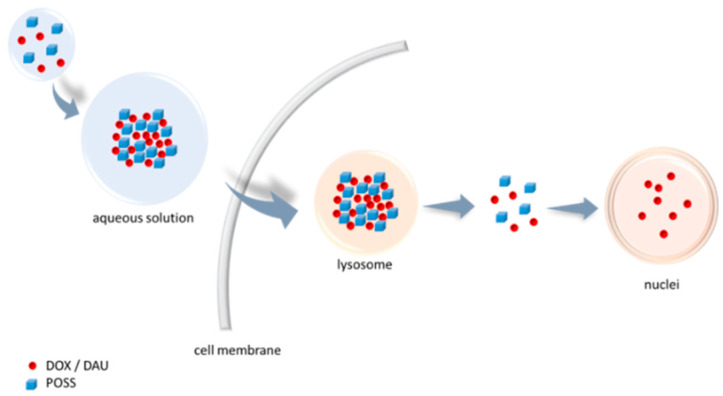
Scheme of co-delivery of a hydroxyl functionalised polyhedral oligomeric silsesquioxane (POSS(OH)_32_) and an anthracycline (e.g. doxorubicin [DOX] or daunorubicin [DAU]) into the cell.

The anthracycline antibiotics doxorubicin (DOX) and daunorubicin (DAU) are anti-cancer drugs that are highly effective in the clinical treatment of a wide variety of tumours. However, administration of anthracyclines can be limited by the occurrence of undesirable side-effects such as cardiotoxicity and inherent drug resistance. Drug delivery via nanocarriers offers an important approach that can improve anti-tumour activity while at the same time decreasing toxicity of anti-cancer drugs towards normal cells [1]. Drugs, conjugated or complexed with nanocarriers (1–100 nm), can easily overcome the barriers constituted by cancer cells’ biological membranes. Such systems improve selective distribution of the active drug and increase the duration of its activity and can thus lead to dose reduction [2]. It is worth stressing that during the last 5–7 years, almost 200 research papers have been published devoted to studies of novel anthracycline (mainly DOX) nanoconjugates and nanocomplexes). So far, many anthracycline nanocarriers such as graphene oxide, poly(lactide-co-glycolide), polyamidoamine dendrimers (PAMAM), and gold have been described [3,4,5,6]. For some time now, we have been studying polyhedral oligomeric silsesquioxanes (POSS) [7] as anthracycline nanocarriers. They are often referred to as the next generation of materials for biomedical applications [8]. Important features of POSS derivatives are their biocompatibility, biodegradability, nanometer size, and lack of toxicity, while the product of their metabolism is a benign orthosilicic acid [9,10,11,12]. During our studies on the synthesis of novel POSS:DOX covalent conjugates [13], we found that simple co-delivery of POSS and DOX led to an unexpected increase in toxicity of the system towards human cervical cancer endothelial (HeLa) and human breast adenocarcinoma (MCF-7) cell lines. Thus, half maximal inhibitory concentration (IC_50_) values for the POSS:DOX complex towards MCF-7 and HeLa cell lines were, respectively, 2.69 ± 0.15 and 0.92 ± 0.09 μM·L^−1^, compared with 17.44 ± 5.23 and 1.45 ± 0.15 μM·L^−1^ for DOX itself. These findings pointed to increased transport effectiveness of DOX to the cells by means of octa(3-aminopropyl)silsesquioxane as the complexing nanocarrier [14]. Similar anti-cancer activity enhancement had been reported earlier by Ohta et al. [15] for the interaction of amino-modified silicon quantum dots (Si-QDs) and DOX. They demonstrated that such species, named Si-QD aggregates, were internalised by HepG2 cells and could function as anti-cancer complexes [15]. In both these reports, the authors proposed NH⋯N hydrogen bonding as a driving force for complex (aggregate) formation; however, at that time, it was only an experimentally unsupported suggestion. Our recent study revealed that not only hydrogen bonding but also electrostatic interactions and π-π stacking between DOX moieties play important roles in the formation of weak complexes [16]. Thus, studies on potential formation of active complexes involving POSS with various functional groups and the anthracycline functional groups also appear generally important for other anti-cancer drug complexing systems, once the presence of required functional groups for such interactions with POSS are identified. The main aim of this work was to expand our findings on other complexes of anthracyclines with hydrophilic silsesquioxane, denoted as POSS(OH)_32_ (Figure 1). The POSS cage can be easily synthesised and possesses a large number of hydroxyl moieties suitable for forming hydrogen bonds with anthracyclines. Judging from the effectiveness of POSS as nanocarriers, one can expect that simple co-addition of silsesquioxanes with anti-cancer drugs can provide a useful pathway that improves the treatment of cancer patients. 

## 2. Materials and Methods

### 2.1. Materials

The synthesis of hydroxyl functionalised silsesquioxanes, referred to in the literature as POSS(OH)_32_, was carried out according to the procedure previously described [17]. These silsesquioxane nanoparticles consist of a mixture of species having 8−18 silicon atoms and are mainly complete and incomplete cage-like structures. The reproducibility of the synthesised structures was proven by three independent synthetic experiments followed by spectroscopic analyses: ^1^H, ^13^C, ^29^Si, ^1^H-^13^C Heteronuclear Single Quantum Coherence (HSQC) and ^1^H-^1^H Correlation Spectroscopy (COSY) NMR, Fourier Transform Infrared Spectroscopy (FTIR) and elemental analysis (see ESI^†^). Hydrochlorides of DOX and DAU were obtained from Sigma-Aldrich (Poznan, Poland). Dimethyl-d_6_-sulfoxide (DMSO-d_6_, 99.8 atom%D, ARMAR, Leipzig, Germany), deuterium oxide (D_2_O, 99.9 atom%D, Sigma-Aldrich, Poznan, Poland) and phosphate-buffered saline (PBS, tablets, Merck, Poznan, Poland) were used as supplied. Complexes of DOX and DAU with POSS(OH)_32_ were prepared by dissolving the anthracyclines in PBS (pH 7.4)/DMSO-d_6_ or D_2_O, followed by addition of POSS solution in relevant solvents. The molar ratio of DOX/DAU: POSS(OH)_32_ was 8:1. All the preparation steps were carried out in oven-dried glassware and under an argon atmosphere. All cell culture reagents were purchased from Gibco (Darmstadt, Germany). Flasks and multi-well plates were obtained from Nunc (Darmstadt, Germany). MTT (3-[4,5-dimethylthiazol-2-yl]-2,5-diphenyltetrazolium bromide) and DOX were supplied by Sigma-Aldrich, Poznan, Poland. Microvascular endothelial (HMEC-1), MCF-7 and HeLa cell lines were obtained from American Type Culture Collection (ATCC, Manassas, VA, USA).

### 2.2. NMR

Two-dimensional nuclear Overhauser effect spectroscopy (2D NOESY) ^1^H NMR spectra were recorded using a 500 MHz Bruker Avance III (Bruker BioSpin GmbH, Rheinstetten, Germany) instrument. Chemical shifts are reported in ppm downfield from TMS using DMSO-d_6_ and D_2_O as solvents (Figures S1, S2 and S4; Supplementary Materials). The 2D NOESY (300 K) analysis was performed in DMSO-d_6_. The complexes were prepared at room temperature with a 5 × 10^−3^ g·mL^−1^ DOX/DAU concentration in DMSO-d_6_. Size of fid (TD): 4096-512; spectral width (SW): 16.0214 ppm (8012.820 Hz); acquisition time (AQ): 0.2555904–0.0319488 s; number of scans: 16; TM1: 0.2-0.1, TM2: 0.7-0.9, delays D1:1 s ^1^H-^1^H COSY analyses were carried out in D_2_O. POSS:DOX and POSS:DAU complexes were prepared at room temperature with 2.22 × 10^−2^ g·mL^−1^ DOX and 2.20 × 10^−2^ g·mL^−1^ DAU, respectively, in D_2_O. Size of fid (TD): 4096-256; spectral width (SW): 10.9942 ppm (5498.534 Hz); acquisition time (AQ): 0.3724629–0.0465579 s; number of scans: 6; TM1: 0-0.1; TM2: 0-0.9; delays D1:1 s.

### 2.3. FTIR

FTIR spectra were obtained using a Nicolet 6700 spectrometer (Thermo Scientific, Spectro-lab Sp. z o.o., Warsaw, Poland) equipped with a deuterated triglycine sulfate detector and attenuated total reflectance (ATR) for 64 scans at a 2 cm^−1^ resolution (Figure S3; Supplementary Materials). Anthracycline complexes with POSS were prepared at an 8:1 molar ratio (incubation: 24 h, 37 °C) and 2.22 × 10^−2^ g·mL^−1^ DOX/DAU in PBS.

### 2.4. Cell Cultures

HMEC-1 cells were grown in an MCDB131 medium supplemented with L-glutamine, hydrocortisone and epidermal growth factor. MCF-7 cells were grown in Minimum Essential Medium (MEM), while HeLa cells were grown in Dulbecco’s Modified Eagle Medium (DMEM). Ten per cent foetal bovine serum (FBS) and streptomycin (100 mg·mL^−1^) were added to all cell culture media. The cells were grown in T-75 culture flasks at 310 K in an atmosphere containing 5% CO_2_. The cells were sub-cultured every 2–3 days. Cell viability was determined by the trypan blue exclusion assay with the use of a Countess Automated Cell Counter (Invitrogen, Carlsbad, CA, USA). Cells were seeded either in 96-well plates at 1.5 × 10^4^ cells/well in 100 µL of an appropriate medium or in 12-well plates at 2.0 × 10^5^ cells/well. After seeding, the plates were incubated for 24 h in a humidified atmosphere containing 5.0% CO_2_ at 310 K in order to allow cells to attach to the plates.

### 2.5. Hydrodynamic Diameter

Hydrodynamic diameters of particles were measured in deionised water at 310 K by dynamic light scattering (DLS) using a Zeta Sizer Nano ZS (Malvern Products, AP Instruments, Warsaw, Poland). Hydrodynamic diameter measurements were performed for pure POSS (50 µM) and for complexes formed by POSS with DOX or DAU at different molar ratios (1:1, 1:4, 1:8, 1:12 and 1:16) immediately after mixing the components and after 24-h incubation. For each sample, measurements were performed three times in three subsequent replications.

### 2.6. Transmission Electron Microscopy

Transmission electron microscopy (TEM) was performed using a Talos F200X electron microscope (Thermo Scientific, Spectro-lab Sp. z o.o., Warsaw, Poland) operating at 200 kV. Anthracycline complexes were prepared by mixing DOX/DAU with POSS in 8:1 ratio (3 h, 300 K) to a final DOX/DAU concentration in PBS of 1 × 10^−4^ g·mL^−1^. Samples were prepared by placing a drop of an PBS solution of the anthracycline’s complexes on the carbon coated copper grid, and then the solvent was evaporated at room temperature.

### 2.7. In Vitro Methods

The cytotoxicity of free drugs and their complexes with POSS at different molar ratios (1:1, 1:4, 1:8, 1:12 and 1:16) was determined by the MTT assay [18] using 1 to 10 µM of DOX or DAU. Cells were incubated for 24 or 48 h. After incubation, the medium was removed and cells were washed with PBS. Next, 50 µL of 0.5 mg·mL^−1^ MTT solution in PBS was added to each well and cells were further incubated under normal culture conditions for 4 h. After incubation, the MTT solution was removed and the obtained formazan precipitate was dissolved in DMSO (100 µL/well). The conversion of the tetrazolium salt (MTT) to a coloured formazan by mitochondrial and cytosolic dehydrogenases was a marker of cell viability. Before the absorbance measurement, plates were shaken for 1 min, and the absorbance at 570 nm was measured with a PowerWave HT Microplate Spectrophotometer (BioTek, Winooski, VT, USA).

### 2.8. Uptake Detection

In vitro uptake studies were carried out using fluorescent DOX or DAU and their complexes with POSS at different molar ratios (1:1, 1:4, 1:8, 1:12 and 1:16). DOX/DAU (1 µM) and their complexes with POSS were added to 12-well plates containing cells (2.0 × 10^4^ cells/well). Cells were incubated with the compounds for a specific period of time (5 min to 48 h) in a humidified atmosphere containing 5.0% CO_2_ at 310 K. After the appropriate incubation period, cells were washed with PBS, suspended in 500 µL of medium and immediately analysed with a Becton Dickinson LSR II flow cytometer (BD Biosciences, San Jose, CA, USA) using a blue laser at 488 nm and phycoerythrin (PE) band pass filter (BD, Franklin Lakes, NJ, USA) at 575/26 nm.

### 2.9. Statistical Analysis

Data are expressed as mean standard deviation (SD). Analysis of variance (ANOVA) with post hoc test (Tukey) was used for multiple comparisons. Statistics were calculated using Statistica software v13.1 (StatSoft, Tulsa, OK, USA), and *p* < 0.05 was considered significant.

## 3. Results

**Figure 2:**
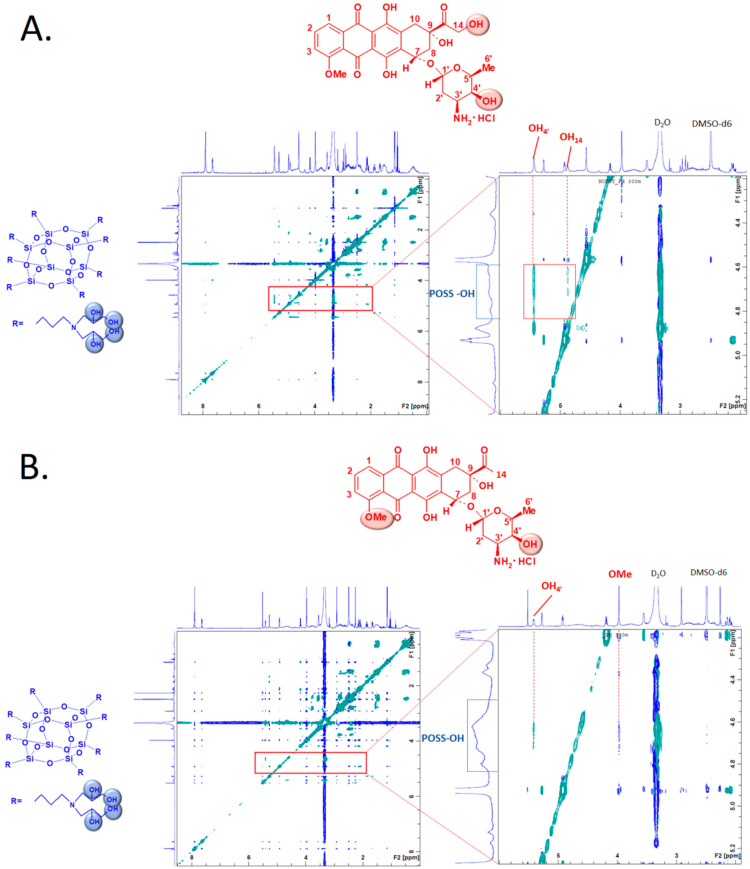

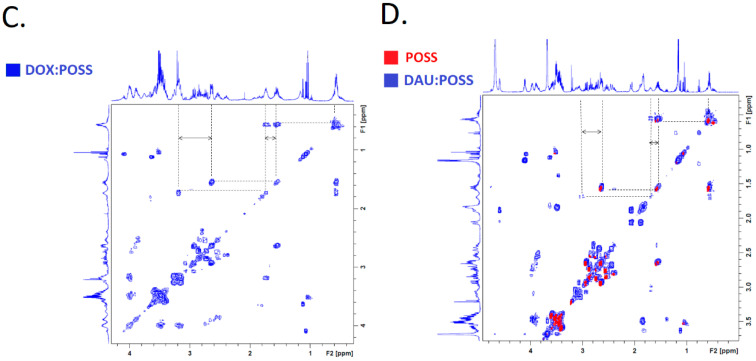
Two-dimensional nuclear Overhauser effect (2D NOESY) spectra of the DOX:POSS (**A**) and DAU:POSS (**B**) complexes in DMSO-d_6_ (300 K, 500 MHz). The homonuclear correlation spectroscopy (^1^H-^1^H COSY) spectra of DOX:POSS (**C**) and DAU:POSS (**D**) complexes, compared with POSS in D_2_O (295 K, 500 MHz). Abbreviations: D_2_O, deuterium oxide; DAU, daunorubicin; DMSO-d_6_, dimethyl-d_6_-sulfoxide; DOX, doxorubicin; POSS, polyhedral oligomeric silsesquioxanes.

The nuclear Overhauser effect (NOE) is an especially useful tool to determine both the structure and dynamics of molecules. The observed NOE signals (using 2D NOESY NMR) for anthracycline:POSS complexes allowed us to determine which hydroxyl groups of POSS and protons of the anthracycline (DOX or DAU) [19] are engaged in the mutual interactions. DOX/DAU:POSS complexes were prepared at an 8:1 molar ratio (Table S1, see ESI†). The 2D NOESY (Figure 2A) spectra of the DOX:POSS complex showed cross-correlation peaks for hydroxyl groups of POSS (~4.3–4.8 ppm) and DOX hydroxyl groups OH_4’_ (~5.4 ppm) and OH_14_ (~4.9 ppm). There were analogous interactions in the 2D NOESY spectrum for the DAU:POSS complex. The protons of POSS hydroxyl groups interact with the OH_4’_ and OMe substituents in DAU. 

The ^1^H-^1^H COSY analysis was run for DOX:POSS, DAU:POSS and POSS alone (Figure 2C,D) to compare the changes in chemical shifts of POSS under the influence of anthracyclines. The results showed the formation of DOX/DAU:POSS complexes that led to changes in resonances originating from POSS protons H_2_ (~1.57 ppm) and H_3_ (~2.65 ppm) towards higher δ values (DOX:POSS [H_2_: ~1.74 ppm, H_3_: ~3.19 ppm] and DAU:POSS [H_2_: ~1.69 ppm, H_3_: ~3.06 ppm]).

**Figure 3:**
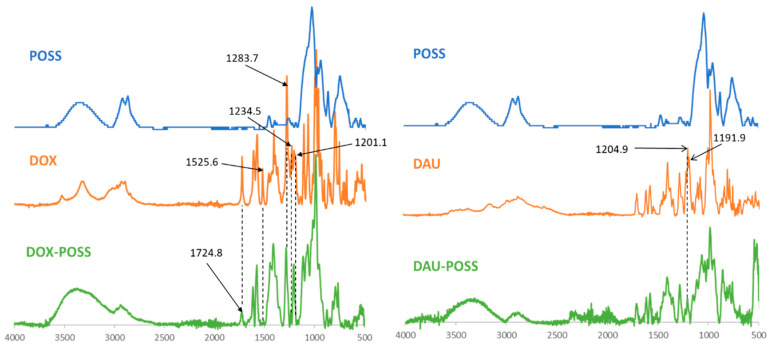
Fourier transform infrared spectroscopy (FTIR) spectra of doxorubicin (DOX), daunorubicin (DAU) and their complexes with polyhedral oligomeric silsesquioxane (POSS).

We used FTIR to study the structural characteristics of the DOX/DAU:POSS complexes in PBS (pH 7.4) after 24 h at 310 K (Figure 3). The FTIR spectrum of DOX indicated the characteristic peaks at 3523 cm^−1^ (ν_O-H_); 3321 cm^−1^ (ν_O-H_, ν_N-H_); 2974, 2954, 2932 and 2896 cm^−1^ (ν_O-H_, aromatic ring), 1724 cm^−1^ (ν_C=O_, ketone); 1616 (sc, NH_2_), 1580 cm^−1^ (ν_C=C_, aromatic ring); 1525 cm^−1^ (δ_C-O-H_, aromatic ring); 1413 cm^−1^ (δ_O-C-H_; δ_N-C-H_); 1283 cm^−1^ (ν_C-O-H_, aromatic ring); 1201 cm^−1^ (ν_C-O_, glycosidic bond); 1234 cm^−1^ (δ_C-O-H_, COCH_2_OH group) and 1205 cm^−1^ (δ_CO-H_, aromatic ring in DAU); and 1191 cm^−1^ (ν_C-O_, glycosidic bond in DAU) [20]. In contrast, the DOX:POSS spectrum indicated that peak intensities at 1525 cm^−1^ (δ_C-O-H_, aromatic ring), 1723 cm^−1^ (ν_C=O_, ketone), 1234 cm^−1^ (δ_C-O-H_, COCH_2_OH group), 1283 cm^−1^ (ν_C-O-H_, aromatic ring) and 1201 cm^−1^ (ν_C-O_, glycosidic bond) decreased. The FTIR spectrum of the DAU:POSS complex (Figure 3) in PBS differed from that of the DOX complex spectrum. Specifically, the intensity of absorption at 1205 cm^−1^ (δ_C-O-H_, aromatic ring) and 1191 cm^−1^ (ν_C-O_, glycosidic bond) decreased in the DAU:POSS complex.

**Figure 4:**
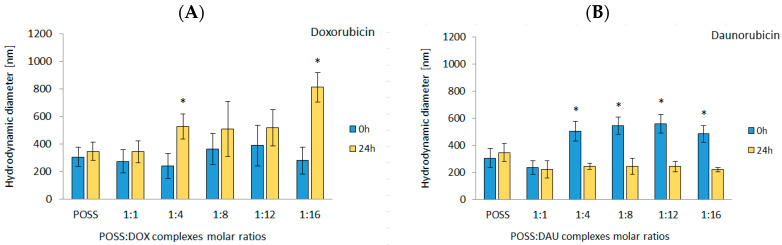
Hydrodynamic diameters of POSS:DOX (**A**) and POSS:DAU (**B**) complexes at different molar ratios after addition of POSS and after 24-h incubation. Data are presented as a percentage of control ± standard deviation (SD); * *p* < 0.05 (statistical significance between POSS and POSS:DOX; POSS:DAU complexes). Abbreviations: DAU, daunorubicin; DOX, doxorubicin; POSS, polyhedral oligomeric silsesquioxanes.

To further confirm the formation of complexes between POSS(OH)_32_ and anthracyclines, we employed DLS to measure the hydrodynamic diameter. The diameter of complexes was measured immediately after the addition of POSS to DOX or DAU and after 24 h. In the case of DOX, the initial diameter of the complexes was similar to that of POSS and did not change as the POSS:DOX molar ratio increased. However, after 24-h incubation at 310 K, the diameters increased. The highest values occurred for 1:16 molar ratio (~800 nm) (Figure 4A). On the contrary, in the case of DAU, the addition of polyhedral oligosilsesquioxanes resulted in an immediate small increase in the hydrodynamic diameter (Figure 4B). After 24-h incubation, there was a decrease in the diameter of the complexes. Importantly, despite the fact that Desai et al. reported that 100 nm nanoparticles exhibited even a 2.5-fold greater uptake compared to 1000 nm diameter microparticles [21] and the hydrodynamic diameter of POSS and most of the complexes exceeded the size of 100 nm, subsequent studies have shown that the use of POSS as a carrier improved the efficiency of transport and anthracycline activity. The observed increase of the hydrodynamic diameter of POSS complexes with DOX can originate from higher number of flat anthracycline molecules that can interact with OH functions of POSS as shown e.g., for the 1:16 complex.

**Figure 5:**
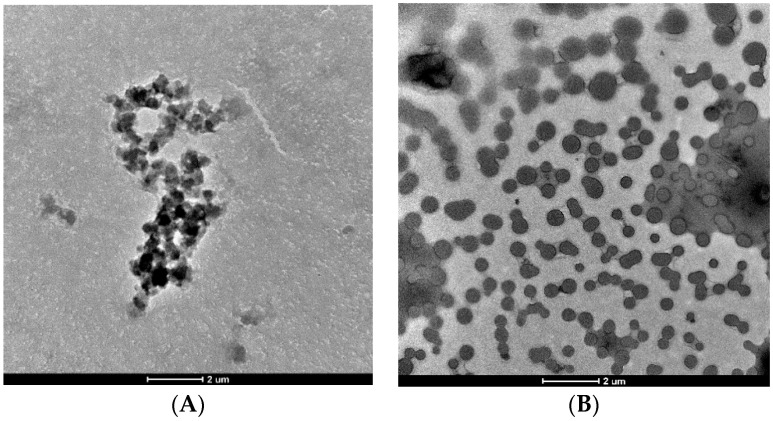
Transmission electron microscopy (TEM) of the POSS:DOX (**A**) and POSS:DAU (**B**) complexes after a 3-h incubation. Abbreviations: DAU, daunorubicin; DOX, doxorubicin; POSS, polyhedral oligomeric silsesquioxanes.

We observed the interaction between DOX/DAU and POSS in PBS using TEM. As shown in Figure 5A, there were 3–5 µM structures formed after 3-h incubation of DOX and POSS. By contrast, in the case of DAU and POSS (Figure 5B), the carrier and drug formed much smaller 100–600 nm structures. The TEM images revealed that DOX interacted stronger with POSS via formation of supramolecular clusters. Although TEM samples bear little resemblance to the samples under solution-based physiological conditions, the results from both methods (DLS and TEM) point to higher stability of POSS:DOX complexes.

**Figure 6:**
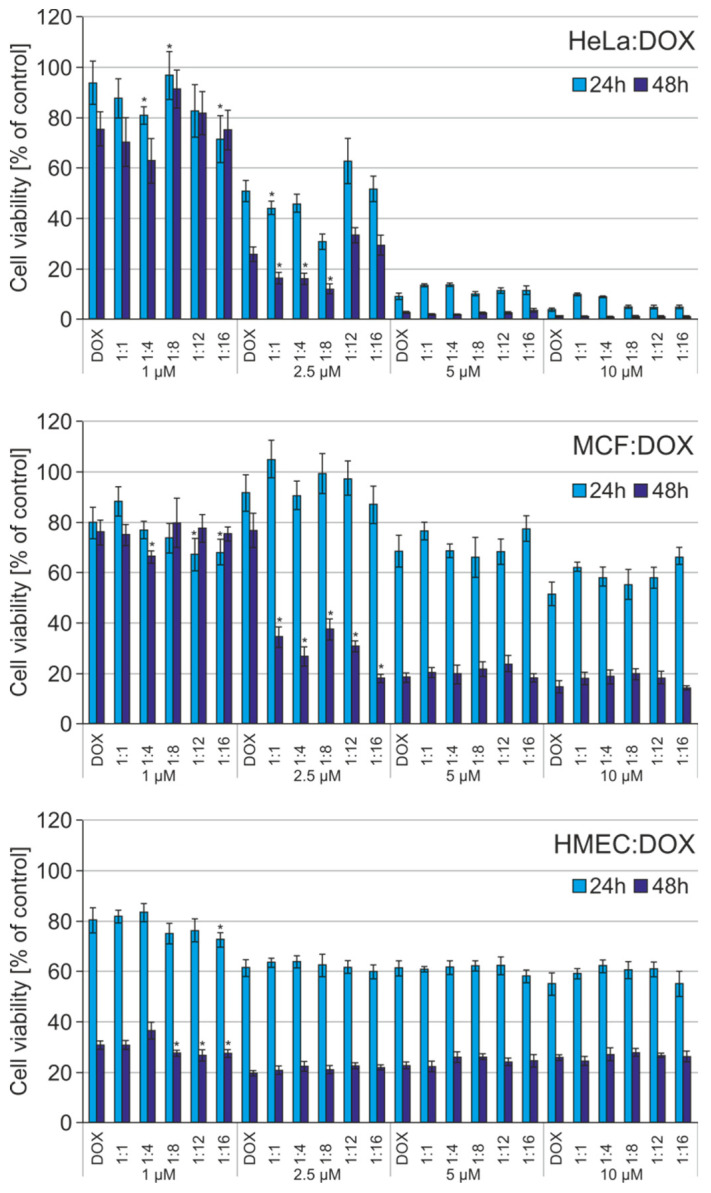
The influence of doxorubicin (DOX) and its polyhedral oligosilsesquioxanes (POSS) complexes at different molar ratios on cell viability of human cervical cancer endothelial (HeLa), human breast adenocarcinoma (MCF-7) and non-cancerous microvascular endothelial (HMEC-1) cells. POSS systems are found to be non-toxic [7]. Data are presented as a percentage of control (untreated cells) standard deviation (SD); * *p* < 0.05 (statistical significance between DOX and POSS:DOX complexes).

Anthracyclines contain flat aromatic moieties that intercalate between DNA bases. DOX binds to DNA and topoisomerase II isoenzymes to form the ternary TopII–DOX–DNA complex, which leads to double-stranded DNA breakage [22]. Even after exposing cells to DOX for 30 min, its presence can be seen in the lysosomes and nuclei [23]. Therefore, in the next step, we examined whether the formation of complexes has an impact on increasing cytotoxicity of the anthracyclines. The viability of HeLa, MCF-7 and non-cancerous HMEC-1 cells was analysed with the MTT assay to evaluate cytotoxicity of free DOX and DAU and the POSS:DOX/DAU complexes at different molar ratios. Cells were incubated with DOX, DAU, POSS:DOX or POSS:DAU for 24 and 48 h with 1–10 µM of the drug. Figure 6 shows the results for DOX and POSS:DOX for both incubation times. As expected, as the DOX concentration increased, there was higher cancer cell mortality. There was a surprising lack of sensitivity to DOX for non-cancerous HMEC-1 cells. In addition, the use of POSS as a carrier did not affect the increase in cytotoxicity in the case of this cell line. 

DOX was more toxic after 48 h, but the use of POSS intensified the cytotoxic effect for all cell lines. The obtained results are consistent with the mechanism of drug action and the time of doubling applied cell lines. After intercalation of DOX in DNA in the cell’s nucleus, the cell must reach one of the checkpoints in the cell cycle, when the repair system directs the cell to programmed death: apoptosis [24]. In HeLa cells, the complexes showed higher cytotoxicity than free DOX after 24 h incubation at lower concentrations, such as 1 µM and 1:4, 1:8 and 1:16 molar ratios; and 2.5 µM at the 1:1 molar ratio. After 48 h incubation complexes showed higher cytotoxicity than free DOX for 1:1, 1:4, and 1:8 molar ratios in HeLa cells. In the case MCF-7 cells, at 2.5 µM all the complexes were more effective than free DOX after 48 h incubation. For 24 h incubation only for 1 µM concentration and for 1:12 and 1:16 molar ratios, the complexes showed higher cytotoxicity than free DOX in MCF cells.

**Figure 7:**
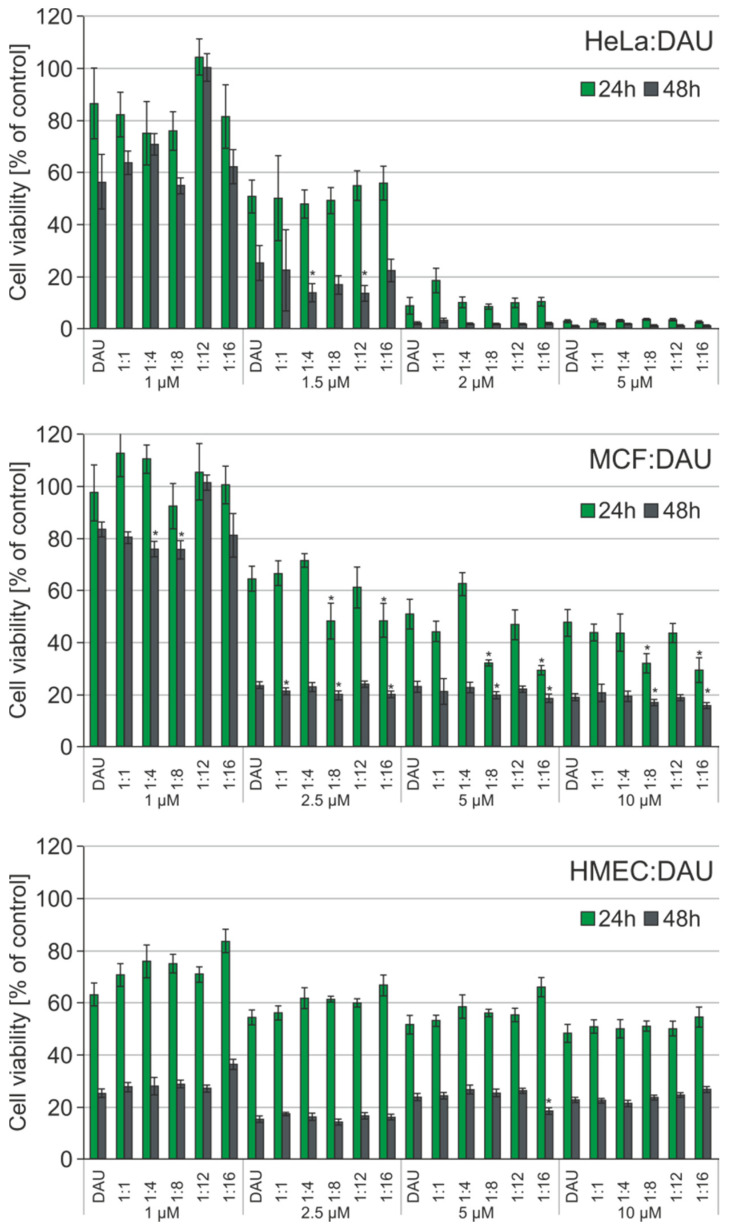
The influence of daunorubicin (DAU) and polyhedral oligosilsesquioxanes (POSS) complexes at different molar ratios on cell viability of human cervical cancer endothelial (HeLa), human breast adenocarcinoma (MCF-7), and non-cancer microvascular endothelial (HMEC-1) cells. POSS systems are found to be non-toxic [7]. Data are presented as a percentage of control (untreated cells) standard deviation (SD); * *p* < 0.05 (statistical significance between DAU and POSS:DAU complexes).

The MTT test using DAU was repeated with the same conditions and analogous complexes. Figure 7 shows the results for free DAU and POSS:DAU complexes for 24- and 48-h incubations. Complexes of POSS(OH)_32_ with DAU at the molar ratios 1:4 and 1:12 and a 1.5 µM DAU concentration showed higher toxicity towards HeLa cells then free drug after 48 h. In the case of the MCF-7 cell line, the improved cytotoxic effect occurred after 24- and 48-h incubation for the 1:8 and 1:16 molar ratios in the 2.5–10 µM concentration range. Similar to DOX, there was a surprising lack of sensitivity towards POSS:DAU complexes by the non-cancer HMEC-1 cell line. 

**Figure 8:**
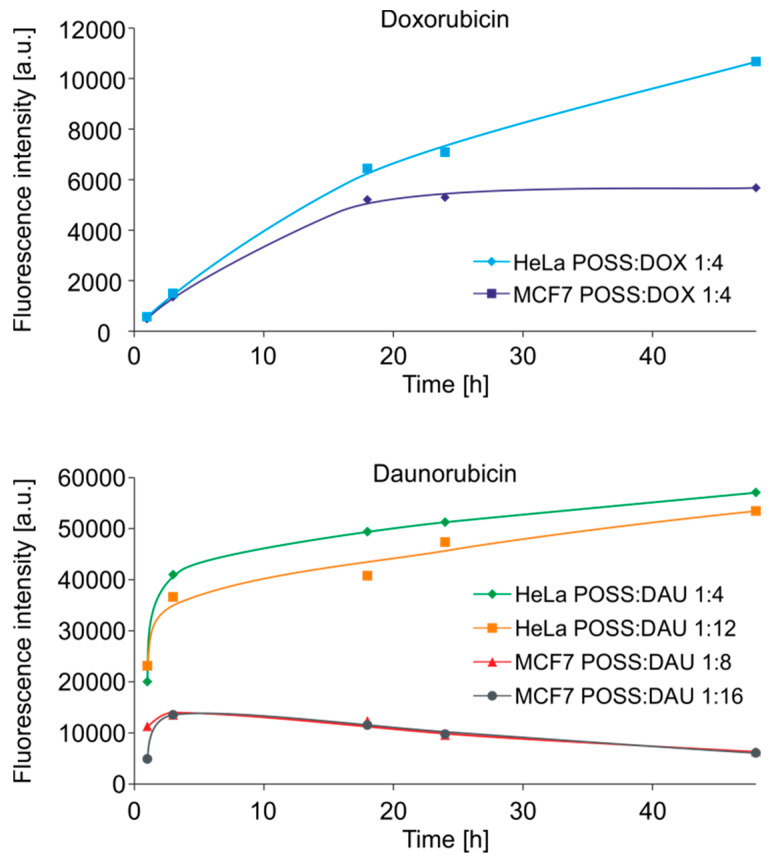
The cellular uptake of selected POSS:DOX and POSS:DAU complexes at different molar ratio by human cervical cancer endothelial (HeLa) and human breast adenocarcinoma (MCF-7) cells. Abbreviations: DAU, daunorubicin; DOX, doxorubicin; POSS, polyhedral oligomeric silsesquioxanes.

The results obtained from hydrodynamic diameter measurements are in good agreement with the results of cytotoxicity assessed with MTT. DOX formed complexes after 24-h incubation and, therefore, their greatest effectiveness occurred after 48 h. In comparison, DAU formed less stable complexes, the effect of which occurred after 24-h incubation. Using the autofluorescence of anthracyclines, we measured the fluorescence of the POSS:DOX and POSS:DAU complexes as a function of time with a flow cytometer in order to confirm the postulated mechanism of action. Figure 8 shows the cellular uptake of the selected most effective complexes by HeLa and MCF-7 cells. As in previous studies, cellular uptake of POSS complexed anthracyclines was higher than that of free drugs [14].

Moreover, as predicted, in the first few hours, there was an increase in the amount of DOX in both the cell lines, with the fastest penetrating complex having a 1:4 POSS:DOX molar ratio (Figure 8, upper panel). In the case of DAU, the cytotoxicity as well as transport rate depended on the cell line. The most toxic to HeLa cells, complexes with a 1:4 and 1:12 molar ratio, were transported at a higher rate to these cells than the most toxic complexes with a 1:8 and 1:16 molar ratio to MCF7 cells (Figure 8, lower panel). In the case of non-cancer HMEC-1 cells, there were no differences in the transport rate to both cell lines (data not shown). The obtained results are consistent and confirm the results obtained by both DLS and MTT methods. 

## 4. Discussion

We demonstrated that POSS can form non-covalent supramolecular complexes with anthracyclines. We showed the formation of such complexes by NOESY NMR analysis, which revealed cross-correlation peaks for POSS hydroxyl groups and OH_4’_ and OH_14_ of DOX, confirming the formation of hydrogen bonds. On the other hand, in the POSS:DAU complex, the OH_4’_ and OMe groups of DAU interact with the POSS hydroxyl groups. In contrast to the POSS:DAU complex, the OMe DOX group did not show clear interaction with POSS in the NOESY analysis. These interactions are likely much weaker compared with the most evident hydrogen bonding with OH_14_ hydroxyl group in DOX. NOESY spectra did not give clear information about possible interactions of POSS with hydroxyl groups at the aromatic ring of anthracyclines (OH_Ar_), because the signals from the OH_Ar_ functions are significantly broadened. 

However, we obtained such information from FTIR analysis because it clearly indicates that there are interactions of anthracycline OH_Ar_ groups with POSS. The decrease in the absorption intensity indicates the interaction of DOX/DAU with POSS via hydrogen bonding between the OH groups in the anthraquinone ring with the POSS OH moieties [14]. Moreover, IR spectra revealed that POSS strongly interacts with DOX via interaction of glycosidic and ketone oxygens and, as mentioned above, the hydroxyl group at carbon 14. In the case of DAU, FTIR revealed hydrogen bonding between the silsesquioxane OH groups and the ethereal oxygen of the glycosidic moiety of daunorubicin. In addition, ^1^H-^1^H COSY NMR revealed chemical shift changes of the resonances originating from POSS protons (H_2_ and H_3_) towards higher δ values, supporting formation of POSS:DOX/DAU complexes in D_2_O.

The decrease in the diameter of the POSS:DAU complexes after 24-h (for DLS) or 3-h (for TEM) incubation may indicate that DAU forms much weaker complexes with POSS(OH)_32_. On the contrary, the diameters of the POSS:DOX complex increased after 24-h or 3-h incubation; these data demonstrated that POSS:DOX complexes are much more stable. This phenomenon has a direct impact on the rate at which the complexes penetrate cancer cells. The more stable POSS:DOX complexes penetrated cells evenly over the first 18 h of incubation. In the case of POSS:DAU complexes, the highest cellular uptake occurred within the first hour of incubation and, interestingly, depended on the cell line. The transport rate was up to fourfold higher for HeLa than for MCF-7 cells. The cytotoxicity results confirmed that POSS facilitated the DOX/DAU transport; therefore, faster penetrating complexes are more toxic than free drugs. The observed differences between cell lines may also indicate a different POSS-mediated transport mechanism and action depending on the type of tumour. HeLa cells have very low p53 functional activity [25] due to constitutive expression of the human papillomavirus (HPV) E6 protein [26] and may be primarily resistant to apoptosis induced by genotoxic stress. The use of POSS as a nanocarrier might overcome the resistance of this cell line and increase its sensitivity to DAU. On the other hand, MCF-7 human breast cancer cells, which contain oestrogen and progesterone receptors, exposed to low doses of anthracyclines may develop multidrug resistance, which is associated with multidrug resistance protein 1 (MDR1), CD44st and nuclear factor kappa-B (NF-κB) messenger RNA (mRNA) and protein expression [27]. Therefore, our findings support the hypothesis that the use of POSS can improve the rate of anthracycline transport to cancer cells and thus can increase their effectiveness by increasing cytotoxicity while reducing the free drug dose.

## 5. Conclusions

Our studies demonstrated that even small structural differences between anthracyclines have a significant impact on their ability to form complexes with POSS-type nanocarriers. Their distribution, cytotoxic activity, and mode of action depend on the cell line. Based on our observations of the effects of POSS on co-delivery with anthracyclines, we can conclude that POSS-type nanocarriers reduce the need for high concentrations of anti-cancer drugs. Therefore, the use of POSS to facilitate anthracycline drug delivery drug seems to be a promising approach in potential future anti-cancer therapy. The novel complexes are inexpensive to prepare, more effective than free drugs at low systemic toxicity. We found POSS(OH)_32_ to be another useful silsesquioxane [16] that increased the efficacy of anthracyclines. These findings leads to the hypothesis that such cage-type silsesquioxanes with various functional groups can serve as effective nanocarriers in drug delivery.

## Supplementary Materials

The following are available online at https://www.mdpi.com/1996-1944/13/23/5512/s1, Elemental analysis of POSS(OH)_32_; Table S1: Description of reaction parameters; Figure S1: ^1^H NMR (500 MHz, 295K) spectrum of POSS(OH)_32_ in DMSO-d_6_, Figure S2: ^1^H NMR (500 MHz, 295K) spectrum of POSS(OH)_32_ in D_2_O, Figure S3: FTIR spectra of POSS(OH)_32_, Figure S4: ^29^Si NMR spectrum of POSS(OH)_32_ (500 MHz, 295K) in D_2_O.
