# Supplementary material for: Hydrophilic Polyhedral Oligomeric Silsesquioxane, POSS(OH)32, as a Complexing Nanocarrier for Doxorubicin and Daunorubicin
Source: Materials (Basel). 2020 Dec 3;13(23):5512. doi: 10.3390/ma13235512 (PMC7730793; doi:10.3390/ma13235512)
Supplement: Supplementary file 1 [file materials-13-05512-s001.pdf]

Hydrophilic Polyhedral Oligomeric Silsesquioxane, POSS(OH)$_{32}$, as a Complexing Nanocarrier for Doxorubicin and Daunorubicin

Kinga Piorecka, Anna Janaszewska, Marta Majkowska, Monika Marcinkowska, Jan Kurjata, Slawomir Kazmierski, Ewa Radzikowska-Cieciura, Bartlomiej Kost, Przemyslaw Sowinski, Barbara Klajnert-Maculewicz and Wlodzimierz A. Stanczyk

Table of Content

Elemental analysis of POSS(OH)$_{32}$
Table S1. Description of reaction parameters
Figure S1. ^{1}H NMR (500 MHz, 295K) spectrum of POSS(OH)$_{32}$ in DMSO-d$_6$
Figure S2. ^{1}H NMR (500 MHz, 295K) spectrum of POSS(OH)$_{32}$ in D_2O
Figure S3. FTIR spectrum of POSS(OH)$_{32}$
Figure S4. ^{29}Si NMR spectrum of POSS(OH)$_{32}$ (500 MHz, 295K) in D_2O

Elemental analysis of POSS(OH)$_{32}$ samples from three independent syntheses under the same reaction conditions: C 42.02 H 8.06 N 5.36; C 42.08 H 8.03 N 5.32 and C 42.08 H 8.02 N 5.38 (theor. C 41.84, H 7.80, N 5.42).

Table S1. Description of reaction parameters.

Drugs	Technique	Concentration of drug [g·ml^{-1}]	Solvent	Molar ratio (drug : POSS)	Reaction/ analysis temperature (K)
**DOX-POSS**	COSY				295
	2D NOESY	$5 \cdot 10^{-3}$	DMSO-d$_6$	8:1	295
	FTIR	$2.22 \cdot 10^{-2}$	H_2O (pH 6.2)	8:1	310
		$7.40 \cdot 10^{-3}$	PBS (pH 7.3)		
**DAU-POSS**	COSY				295
	2D NOESY	$5 \cdot 10^{-3}$	DMSO-d$_6$	8:1	310
	FTIR	$2.22 \cdot 10^{-2}$	H_2O (pH 6.2) or PBS (pH 7.3)	8:1	310

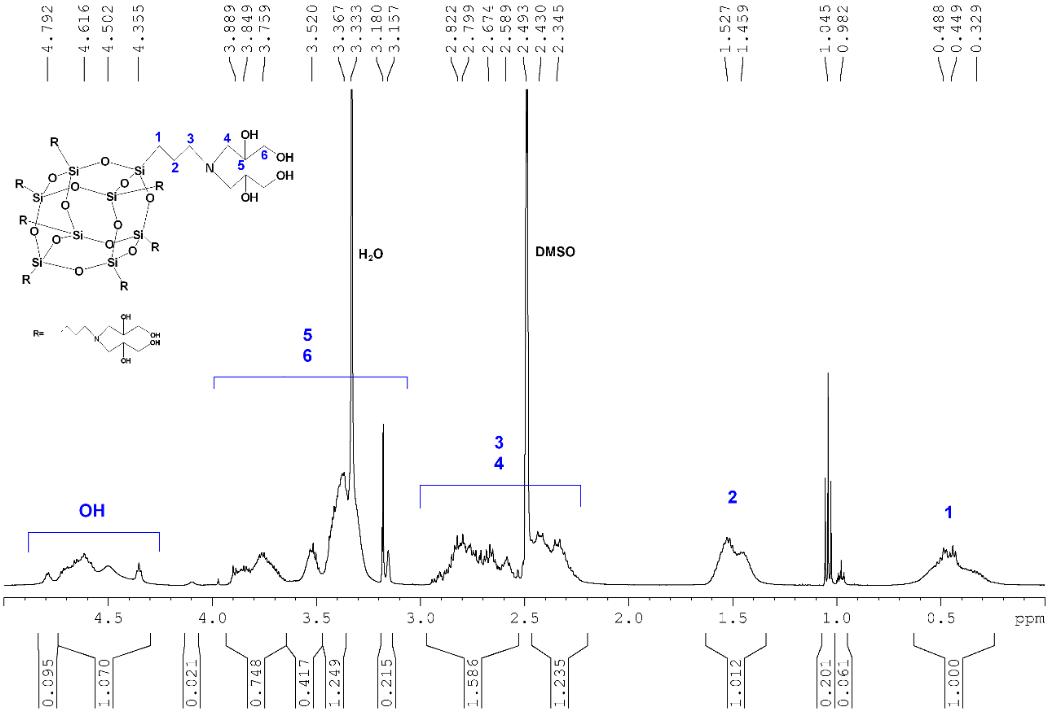

Figure S1. ^{1}H NMR (500 MHz, 295K) spectrum of POSS(OH)$_{32}$ in DMSO-d$_6$.

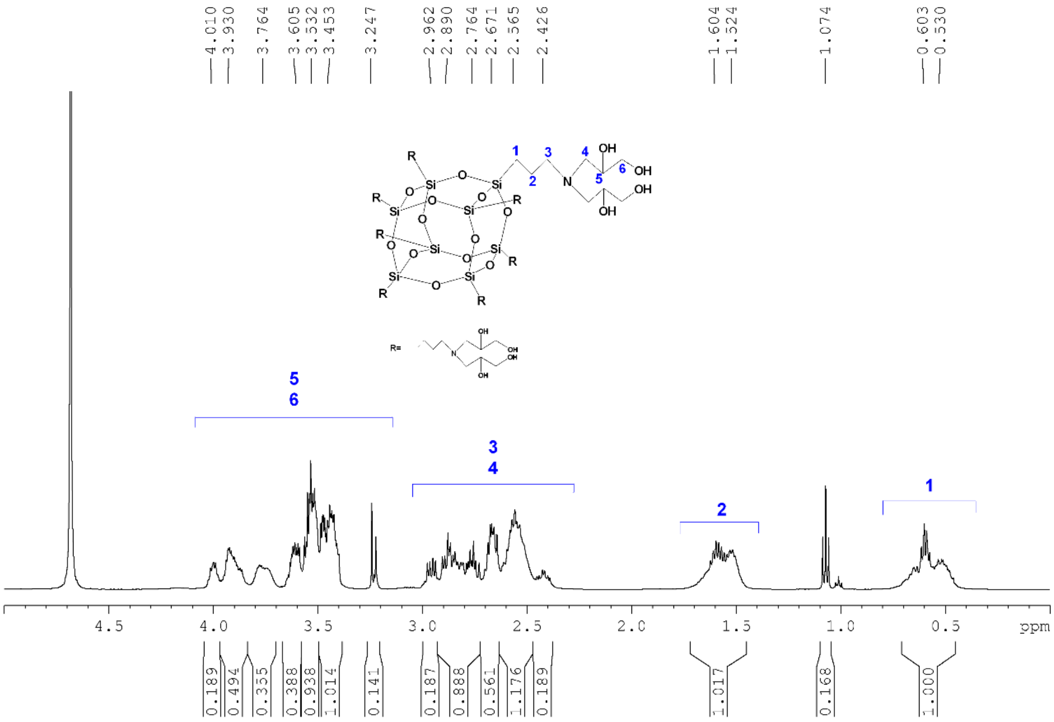

Figure S2. ^{1}H NMR (500 MHz, 295K) spectrum of POSS(OH)$_{32}$ in D_2O.

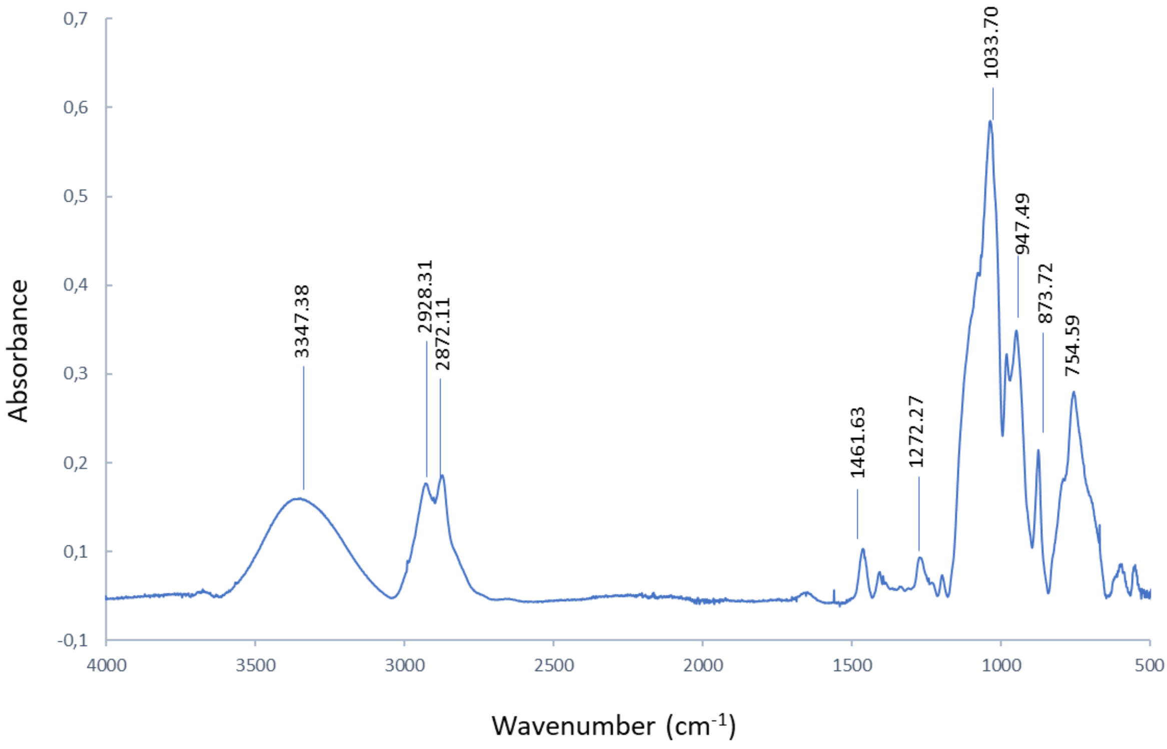

Figure S3. FTIR spectrum of $POSS(OH)_{32}$.

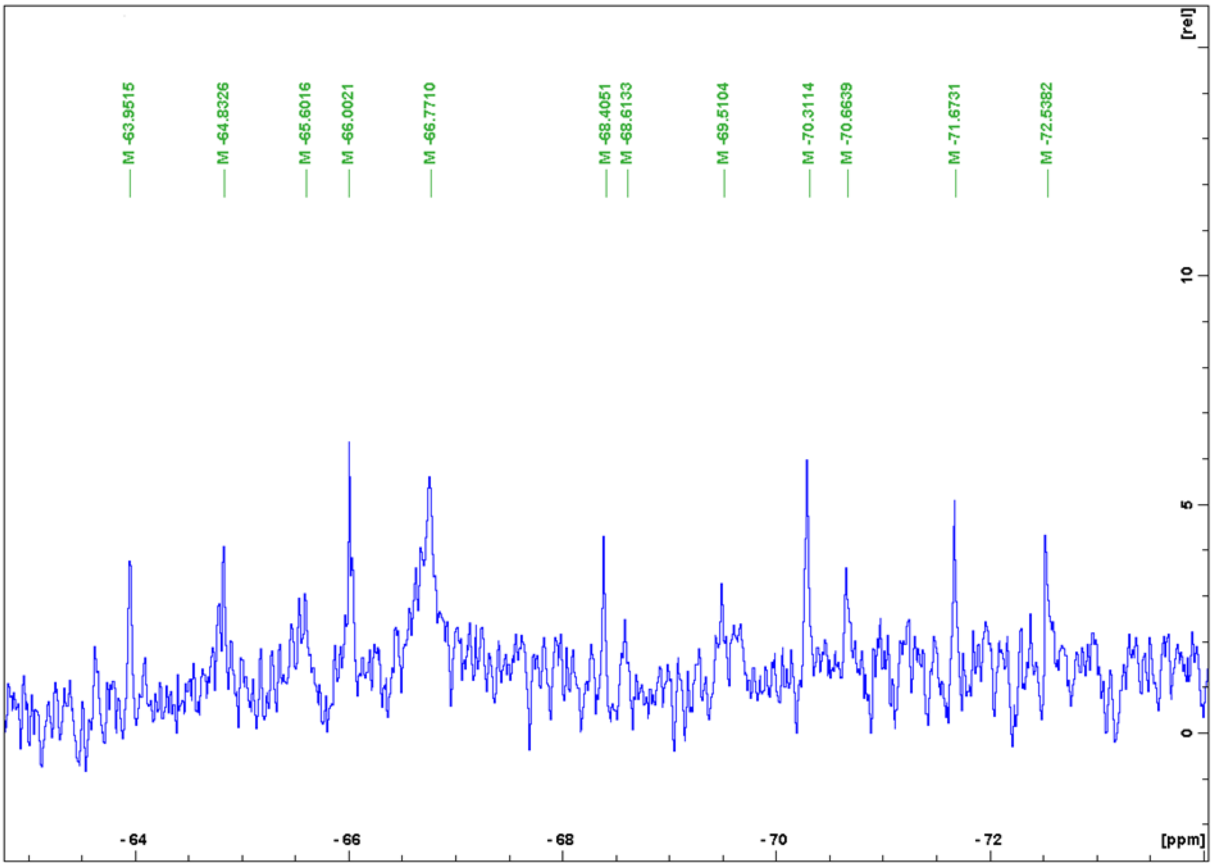

Figure S4. ^{29}Si NMR spectrum of $POSS(OH)_{32}$ (500 MHz, 295K) in D_2O.
